# Effectiveness of measures taken by governments to support hand hygiene in community settings: a systematic review

**DOI:** 10.1136/bmjgh-2025-018929

**Published:** 2025-09-16

**Authors:** Jedidiah S Snyder, Erika Canda, Jordan Honeycutt, Lilly A O’Brien, Hannah K Rogers, Oliver Cumming, Joanna Esteves Mills, Bruce Gordon, Marlene K Wolfe, Bethany A Caruso, Matthew C Freeman

**Affiliations:** 1 Gangarosa Department of Environmental Health, Emory University, Atlanta, Georgia, USA; 2 Woodruff Health Sciences Center Library, Emory University, Atlanta, Georgia, USA; 3 Department of Disease Control, London School of Hygiene and Tropical Medicine, London, UK; 4 Water, Sanitation, Hygiene and Health Unit, World Health Organization, Geneva, Switzerland; 5 Hubert Department of Global Health, Emory University, Atlanta, Georgia, USA

**Keywords:** Global Health, Hygiene, Systematic review

## Abstract

**Introduction:**

This systematic review aimed to identify and evaluate the implementation of government measures that support equitable and sustained hand hygiene practices in community settings.

**Methods:**

We systematically searched 12 databases, including PubMed, Web of Science, EMBASE, CINAHL, Global Health, Cochrane Library, Global Index Medicus, Scopus, PAIS Index, WHO IRIS, UN Digital Library and World Bank eLibrary for peer-reviewed and grey literature published through late March 2023. Additional sources were identified through expert consultations and manual reference list checks of related reviews. Studies employing quantitative, qualitative or mixed-methods designs were eligible. Study quality was assessed using the Mixed Method Appraisal Tool. Government measures were categorised according to the Sanitation and Water for All Building Blocks framework: sector policy strategy; institutional arrangements; sector financing; planning, monitoring, review; and capacity development. Hand hygiene outcomes were classified as access, behaviour change or enabling environment and impact as positive, null or not evaluated.

**Results:**

Thirty-one studies (24 journal articles and 7 grey literature) from 19 countries—mostly middle income (71%)—were included. Most focused on household (58%), schools (19%) or both (13%). A total of 75 government measures were identified, with sector policy strategy and capacity development being the most common (each 31%), followed by institutional arrangements (17%), planning, monitoring, review (13%) and sector financing (8%). Positive impacts were linked to 45 measures across all five Building Blocks in 17 studies.

**Conclusion:**

This systematic review highlights diverse government measures supporting hand hygiene in community settings, with sector policy strategy and capacity development being the most frequently reported. While many government measures showed positive impacts, gaps remain in financing, implementation and sustainability beyond households and schools. Strengthening governance, increasing investment and expanding research on cost-effectiveness and implementation barriers are essential to improve hygiene initiatives and ensure equitable access.

**PROSPERO registration number:**

CRD42023429145.

**WHAT IS ALREADY KNOWN ON THIS TOPIC**

- Hand hygiene is crucial for preventing infectious diseases, but there is a lack of consistent global guidelines and evidence-based recommendations for government measures in community settings.
- Most existing hand hygiene guidance focuses on healthcare settings, with limited attention to community contexts such as homes, schools and public spaces.

**WHAT THIS STUDY ADDS**

- This systematic review is the first to comprehensively assess government measures supporting hand hygiene in community settings, synthesising evidence across 31 studies from 19 countries.
- It identifies 75 distinct government measures mapped across the Sanitation and Water for All Building Blocks, providing a structured framework to evaluate policy and programmatic action.
- The review highlights key trends, including the predominance of measures related to integrating hand hygiene into broader health programmes and capacity development, mixed evidence on implementation effectiveness and gaps in financing and in public or institutional settings.
- It also underscores challenges in evaluating complex government interventions due to inconsistent implementation details and outcome reporting.

**HOW THIS STUDY MIGHT AFFECT RESEARCH PRACTICE OR POLICY**

- By organising evidence around a widely used systems framework, this study provides a practical insight for governments and global health practitioners on integrating hygiene into broader national strategies, encouraging multisectoral coordination and investment.
- For researchers, it highlights the need for improved reporting on the evaluation of government interventions and greater focus on under-researched settings such as markets, transportation hubs and workplaces.
- This evidence will directly inform the forthcoming WHO Guidelines for Hand Hygiene in Community Settings and help shape global and national efforts to strengthen hygiene systems.

## Introduction

 Poor hand hygiene within households is estimated to contribute to 37 million disability-adjusted life years and over 730 000 yearly deaths annually from diarrhoea and acute respiratory infections—a figure that has remained largely unchanged since 2010.^1^ Interventions to promote hand hygiene, either through soap and water or alcohol-based handrubs (ABHR), are relatively inexpensive to implement^2^ and can prevent several infectious diseases, including enteric^3^ and respiratory^4^ infections, which account for a large burden of disease^5^ and high healthcare costs.^6^ Establishing global guidelines and recommendations is essential to guide hand hygiene initiatives, protect public health and strengthen resilient health systems.^7^

Governments play a central role in creating an enabling environment for effective and sustained hand hygiene through creating policy and funding initiatives. Their leadership is crucial not only in ensuring access to hygiene products and services but also in coordinating across sectors, mobilising resources, engaging communities and shaping national strategies that prioritise hand hygiene. This encompasses a wide range of activities, including legislation, regulations, funding mechanisms, infrastructure development, public education campaigns and strategies for monitoring and enforcement. These may include efforts such as promoting local soap production and fostering public–private partnerships for handwashing,^8 9^ cross-sectoral collaboration for hand hygiene,^9 10^ engaging communities and the private sector for the delivery of services^11 12^ and supporting or reinforcing existing monitoring systems in line with global hygiene indicators.^9 13^ Community settings—where people live, learn, work and interact—play a vital role in shaping health outcomes.^14^ These include domestic, public and institutional spaces.^15^ While hand hygiene guidelines in healthcare settings are well established,^16^,^19^ guidance for community settings remains limited. Although recent global strategies emphasise the importance of investing in hand hygiene,^1120^,^22^ and the COVID-19 pandemic brought renewed urgency to this issue,^11^ guidance on specific hand hygiene measures outside of healthcare settings remains inconsistent and incomplete.^23^ A recent scoping review of 51 international guidelines found limited evidence-based recommendations for community settings, highlighting four key areas where clarity is needed: (1) the effectiveness of various hand hygiene practices to reduce pathogens; (2) the minimum requirements for infrastructure, products and services needed to conducting effective behaviours; (3) effective behaviour change promotion strategies; and (4) information on government measures, including policies and strategies.^15^

This systematic review is part of a collection of related reviews designed to address these identified priority areas and focuses specifically on government measures—defined as initiatives and interventions taken by governments to support hand hygiene to support hand hygiene in community settings.^24^ While a range of hand hygiene initiatives have been implemented globally, MacLeod *et al* found that only 11 of the 51 international guidelines reviewed included specific recommendations on government measures for hand hygiene in community settings. Existing reviews of government measures related to hand hygiene, implemented at scale, are limited to COVID-19 response,^25^ with no systematic reviews evaluating such measures globally within community settings. This review aims to fill that gap by synthesising evidence on the implementation of government measures designed to promote hand hygiene in community settings.

The priority questions for this review were generated through an extensive consultation process by the WHO with external experts,^24 26^ building on a prior scoping review of international guidelines.^15^ Findings from this review, along with related reviews,^27^,^30^ will inform the forthcoming WHO Guidelines for Hand Hygiene in Community Settings. To enhance the synthesis and generalisability across diverse contexts, we applied the Sanitation and Water for All (SWA) Building Blocks framework,^31^ which outlines five key elements of a robust water, sanitation and hygiene (WASH) system: sector policy strategy; institutional arrangements; sector financing; planning, monitoring, review; and capacity development. This shared framework allows for clearer categorisation and comparison of government measures across studies and settings.^32^

This systematic review identifies and synthesises government measures that support equitable and sustained hand hygiene practices in community settings. The findings provide valuable insights into current approaches and offer a foundation for future policy development, programme implementation and global guidance.

## Methods

### Research questions

This systematic review assessed the following questions related to government measures implemented to support equitable and sustained hand hygiene: (a) What government measures have increased access to soap for hand hygiene? (b) What government measures have increased access to water for hand hygiene? (c) What government measures have resulted in changes to end-user hand hygiene practices? (d) Where have governments intervened to address equality and/or affordability of handwashing? and (e) Where have governments intervened to address other intermediate outcomes that could impact end-user access or practices (ie, enabling conditions related to questions a–c), but that did not measure soap access, water access or end-user practices? Across questions a–c, we sought to assess if measures were equitable and sustained.

### Search strategy

This review was preregistered with PROSPERO (registration number: CRD42023429145) and is reported in accordance with the Preferred Reporting Items for Systematic Reviews and Meta-Analyses (PRISMA) criteria^33^ (online supplemental material S1—PRISMA checklist). This review was part of an integrated protocol for multiple related reviews to synthesise the evidence for effective hand hygiene in community settings.^24^ We adopted a two-phased approach for identifying relevant studies. Phase 1 involved a broad search to capture all studies on hand hygiene in community settings that were relevant across multiple related systematic reviews. The outcome of phase 1 was a reduced sample from which further screening, specific to this review, was performed. A full description of the procedures followed for searches, study inclusion, outcomes data collection, analysis and reporting of the multiple related reviews is presented in the published protocol.^24^

This search included studies published through late March 2023, which were either fully in English, had the title and abstract published in English, or was a non-English article cited in an existing systematic review. We searched 12 peer-reviewed and grey literature databases. PubMed, Web of Science, EMBASE (Elsevier), CINAHL (EBSCOhost), Global Health (CAB), Cochrane Library, Global Index Medicus, Scopus (Elsevier), Public Affairs Information Service (PAIS) Index (ProQuest) were searched on 23 March 2023 and WHO Institutional Repository for Information Sharing, UN Digital Library and World Bank eLibrary were searched on 28 March 2023 using search terms related to hand hygiene broadly and restrictions on terms related to healthcare settings in the titles. We searched trial registries (International Clinical Trials Registry Platform and clinicaltrials.gov) for trials related to hand hygiene in community settings on 29 March 2023.

We conducted manual searches of reference lists of two relevant reviews.^15 25^ For reviews that provided a list of the reviewed articles, we searched only for those references. If a list was not available, we searched all references and screened for potentially relevant titles. These reviews had 93 total references of which 4 were duplicates, 2 were already identified in our database search, 51 were identified as non-primary research (eg, guidelines) included in MacLeod *et al*, and 36 were added to phase 2 title and abstract screening. We contacted 35 content experts and organisations, using snowballing methods, between April and May 2023 for information on relevant unpublished literature.

### Selection criteria

Eligibility criteria were based on characteristics describing the studies’ sample, phenomenon of interest, design, evaluation and research type^34^ and are published in our protocol.^24^

Hand hygiene is defined as any hand cleansing undertaken for the purpose of removing or deactivating pathogens from hands, and efficacious hand hygiene is defined as any practice, which effectively removes or deactivates pathogens from hands and thereby has the potential to limit disease transmission.^17^ Community settings are defined as settings where ‘health is created and lived by people within the setting of their everyday life; where they learn, work, play and love’^14^ and include domestic (eg, households), public (eg, markets, public transportation hubs, vulnerable populations (eg, people experiencing homelessness), parks, squares or other public outdoor spaces, shops, restaurants and cafes) and institutional (eg, workplace, schools and universities, places of worship, prisons and places of detention, nursing homes and long-term care facilities) spaces.^15^ Studies were excluded if they were in healthcare settings or were animal research. Studies focusing on care provider’s hand hygiene in nursing homes and long-term care facilities were excluded as part of phase 2 screening as the evidence they generated was determined to be similar to that from healthcare settings. There were no geographic restrictions.

Eligible studies were quantitative, qualitative or mixed methods studies of general populations in community settings and included policy documents and grey literature published after 1 January 1980. Studies were included if the topic of research was on measures taken by governments to ensure effective hand hygiene including government-led initiatives and interventions to increase access to soap for hand washing with soap; ensure access to water for handwashing; deliver behaviour change interventions for promoting handwashing with soap at key moments or address equality and/or affordability.

We used Covidence software for systematic reviews.^35^ In both phases, screening of each article (phase 1—title and abstract only; phase 2—title and abstract, then full-text review) was performed independently by two reviewers, with discordance between reviewers reconciled by a third reviewer. Studies reporting on the same government programme, campaign and/or initiative were grouped together, and the study describing the main evaluation was included for data extraction. The stages of the search and screening process are described in online supplemental material S2—PRISMA flowchart.

### Data analysis

Two reviewers (EC and JH) independently extracted data using a customised data extraction tool and assessed risk of bias for each article using the Mixed Method Appraisal Tool (MMAT).^36 37^ The MMAT assessment results are provided in online supplemental material S3—MMAT Assessment. Qualitative and quantitative studies were assessed using the five-criteria questionnaire. Mixed method studies were assessed using the relevant independent questionnaires for qualitative and quantitative work and a five criteria questionnaire for mixed methods; the lowest of the three scores was used as the quality score. Possible scores are 0–5 across study types (5=highest quality). Any conflicts between reviewers over data extraction and quality assessment were resolved by a third reviewer (JSS).

For each study, identified government measure(s) supporting hand hygiene practices in community settings were categorised according to the SWA Building Blocks, which defines five key elements for a sustainable WASH sector: (1) sector policy strategy; (2) institutional arrangements; (3) sector financing; (4) planning, monitoring, review; and (5) capacity development.^31^ The SWA Building Blocks were chosen for our analysis due to their strong alignment with elements found in other established WASH systems frameworks. While the SWA Building Blocks provide a comprehensive structure for evaluating government interventions, alternative frameworks could have also been considered.^38^,^41^ For example, the WHO Health System Building Blocks framework focuses on broader health system strengthening but lacks the WASH-specific emphasis necessary for this review.^41^ Similarly, the UN-Water SDG 6 Acceleration Framework^39^ and UN-Water Global Analysis and Assessment of Sanitation and Drinking-Water^38^ provide a high-level approach to improving water and sanitation governance but does not focus specifically on hand hygiene promotion. The IRC WASH Systems Building Blocks^40^ emphasises long-term systems change and sustainability but may not fully capture the diversity of policy-driven interventions across different governance structures. Each of these frameworks has strengths, but we selected the SWA Building Blocks due to their established use in evaluating WASH governance, their practical alignment with global policy strategies and their applicability to a broad range of government-led interventions. Furthermore, the SWA Building Blocks have demonstrated practical applicability through their successful integration into AMCOW’s Africa Sanitation Policy Guidelines,^42^ reinforcing their relevance and effectiveness in supporting WASH sector sustainability across diverse contexts.

The studies were qualitatively coded in MAXQDA^43^ to identify programmatic and policy-relevant Building Blocks, using definitions summarised in table 1. Information on the level of the measure (eg, national, regional, provincial, district), the enacting government bodies (and implementing partners) and the targeted populations and community settings were extracted as reported. Details regarding equality and affordability were also noted when available. The target of the measure in relation to hand hygiene was categorised into: (1) access (the measure aimed to increase access to handwashing facilities, soap, water and/or ABHR), (2) behaviour change (the measure aimed to increase changes to end-user hand hygiene practices) and/or (3) enabling environment (the study explicitly noted improving the enabling conditions when describing the measure). For each study, the impact of the measure(s) in relation to hand hygiene outcome of interest was categorised as positive, null or not evaluated, as described in the Results section of the included study. If sustainability of measures with a positive impact was explicitly stated, these details were extracted from the Results and/or Discussion sections. Countries were categorised according to WHO regions and World Bank’s income classifications.^44^

**Table 1:** Definitions of the ‘Building Blocks’^31^ used to categorise measures taken by governments to support hand hygiene in community settings

Building block	Definition
Sector policy strategy (SPS)	Policies and strategies that identify goals and pathways, giving direction to sector investments. Policies and strategies that cover subsectors (urban, rural, drinking water and sanitation) and national/subnational levels. Strategies for implementation, including agreement on implementation models/sustainable service delivery approaches.
Institutional arrangements (IA)	Identification and allocation of institutional roles and responsibilities, including decentralisation commitments. Country-driven coordination mechanisms that allow for participation of a broad range of stakeholders in dialogue, communication and identification of mutual interest around service delivery and sector learning. Legal and regulatory frameworks to underpin the desired targets and reinforce roles and allocation of resources.
Sector financing (SF)	Expenditure framework which matches government priorities with available resources. Realistic and transparent sector budget with identifiable funding streams. Availability and use of data on financing streams including taxes, tariffs and transfers and comparable estimates for cost categories.
Planning, monitoring, review (PMR)	Planning, monitoring and evaluation of sector performance. Mid-and longer-term review of sector performance through multi-stakeholder platforms and mechanisms for sector dialogue and learning. Clearly defined accountability mechanisms. Data transparency and public access to information.
Capacity development (CD)	Capacity building and development plans addressing the capacity of: Institutions to fulfil sector roles and responsibilities for sustainable service delivery at scale, including the availability of necessary structures, tools, training and incentives. Sector stakeholders to adapt and innovate by engaging in sector learning. Individuals to engage in the sector through sector institutions or as educated consumers.

A narrative synthesis of all studies is presented, which includes data-driven descriptive themes of government measures categorised into the five Building Blocks. Due to the scientific and methodological differences across study designs, populations and outcome measures, we did not conduct a statistical meta-analysis. Instead, we used vote counting to explore patterns across data-driven descriptive themes of government measures, specifically categorising findings as positive, null or not evaluated.^45 46^ This approach allowed us to summarise the direction of effects across a diverse evidence base. To support the interpretation of these patterns, we also present the quality of studies in each category using the MMAT assessment, helping to contextualise the strength and reliability of the reported findings.

### Ethics and patient involvement statements

As this is a review of published documents, no ethical approval was required. Patients or the public were not involved directly in the design, conduct, reporting or dissemination plans of our research. This evidence synthesis supports the forthcoming WHO Guidelines for Hand Hygiene in Community Settings, which developed the study questions in broad consultation with key partners and networks of partners.

## Results

### Characteristics and quality of included studies

**Figure 1:**
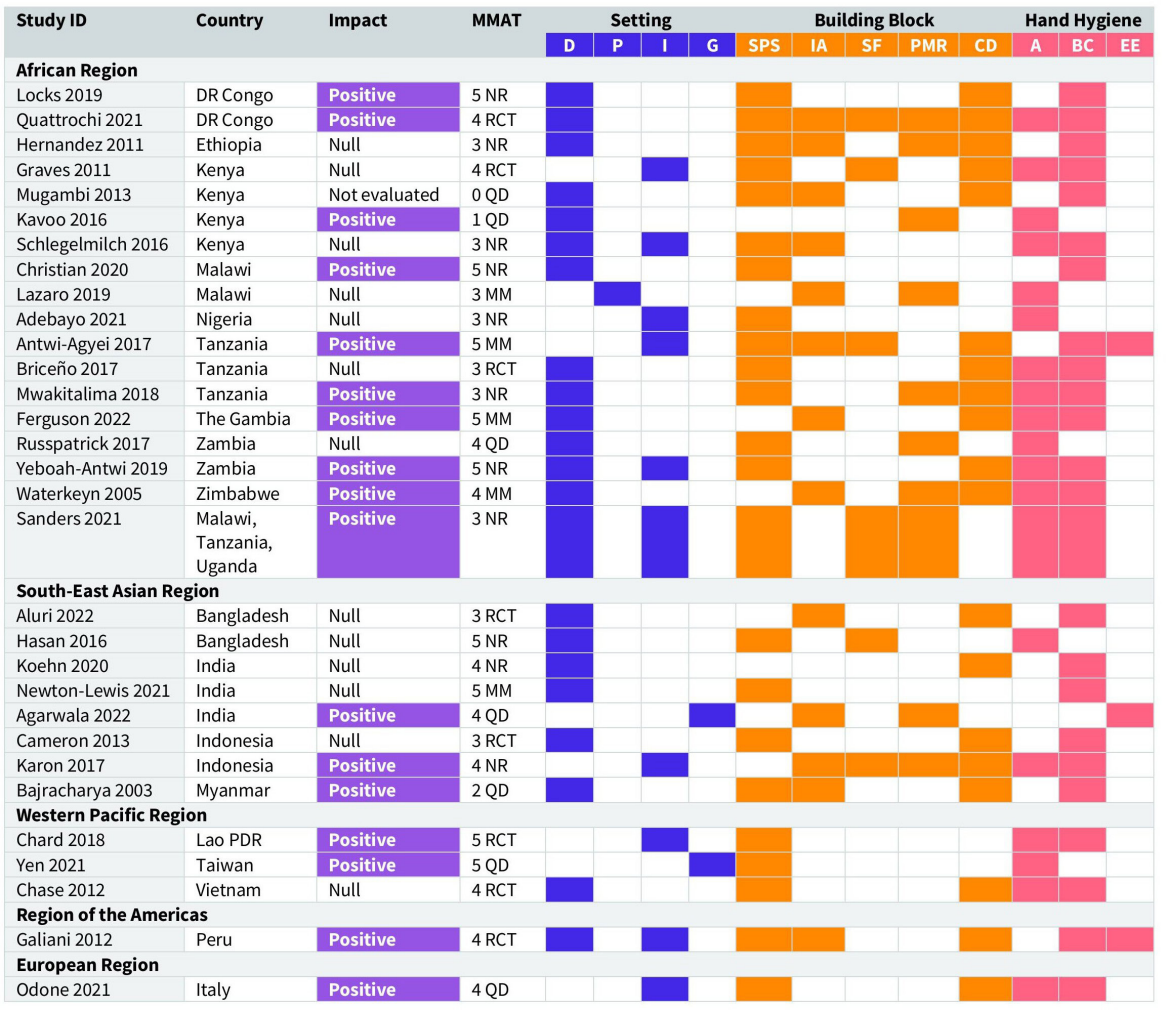
Overview of included studies. More detailed descriptions of each study and government measures are provided in the online supplemental material. This figure summarises each study (N=31) by community setting, Sanitation and Water for All (SWA) Building Blocks addressed, targeted hand hygiene outcomes, reported impact and Mixed Methods Appraisal Tool (MMAT) quality appraisal score. Studies are organised by region and in alphabetical order by country. Impact was categorised as positive, null or not evaluated, based on findings reported in the study results. Quality appraisal used the MMAT (Pluye and Hong^36^; Hong *et al*^37^), with scores ranging from 0 (lowest) to 5 (highest). Study types include NR–non-randomised study, RCT–randomised controlled trial, MM–mixed methods study and QD–quantitative descriptive study. Community setting codes: D–domestic, P–public, I–institutional, G–general community settings. SWA Building Block codes: SPS–sector policy strategy, IA–institutional arrangements, SF–sector financing, PMR–planning, monitoring, review, CD–capacity development. Targeted hand hygiene outcome codes: A–government measure targeting access to facilities/soap/water/ABHR, BC–government measure targeting behaviour change, EE–government measure targeting enabling environment conditions.

We identified 31 studies that met our inclusion criteria, including 24 journal articles and 7 studies published in grey literature (online supplemental material S2—PRISMA flowchart). All identified studies were published in English. Figure 1 provides an overview of each included study, summarising the community setting, SWA Building Blocks addressed, targeted hand hygiene outcomes, reported impact and quality appraisal score. Table 2 presents key study characteristics, including geographic distribution, income level, target settings and outcome focus. A detailed summary of the study characteristics is provided in online supplemental material S4—Summary of Studies and descriptions of the government measures identified across the included studies are provided in online supplemental material S5—Description of Government Measures.

**Table 2:** Characteristics of the included studies

Descriptive characteristics of studies	N (31)	%
Source		
Journal article	24	77%
Grey literature	7	23%
Number of countries represented by the review	19	
Income level*		
High income	2	6%
Upper-middle income	3	10%
Lower-middle income	19	61%
Low income	6	19%
Multi-country	1	3%
Target community setting of government measure		
Household	18	58%
Schools	6	19%
Households and schools	4	13%
Open-air markets	1	3%
General (unspecified)	2	6%
Target areas of government measure		
Rural areas	16	52%
Urban areas	1	3%
Mixed areas	4	13%
General (unspecified)	10	32%
Target hand hygiene outcome of government measure		
Change end-user hand hygiene practices	24	77%
Increase access to hand hygiene facility (soap and water)	15	48%
Increase access to alcohol-based hand rubs	2	6%
Increase access to water availability for hand hygiene	2	6%
Create enabling conditions for hand hygiene	3	10%

* Categorised according to World Bank’s income classifications.^44^

**Figure 2:**
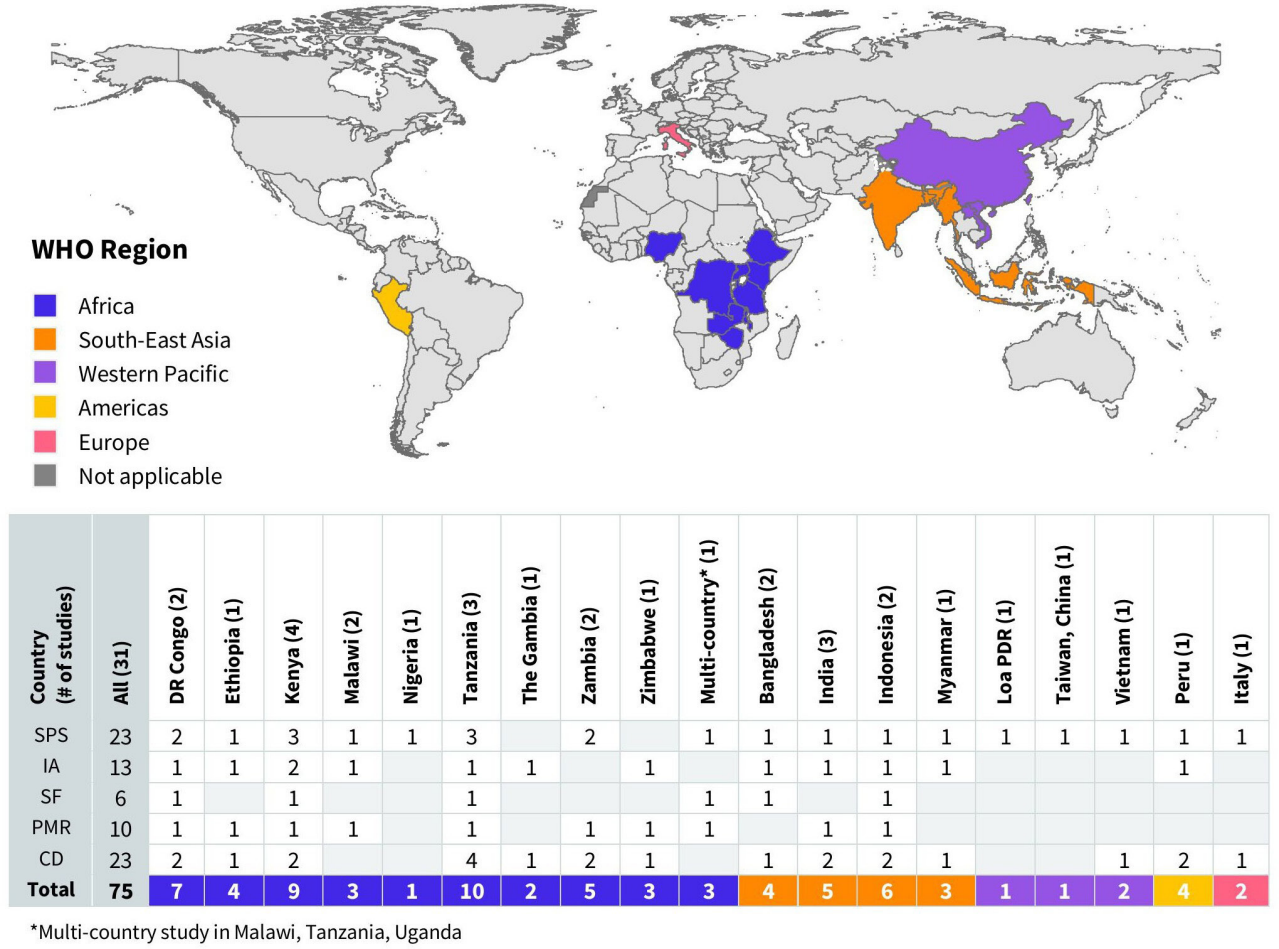
Global distribution of included studies assessing government measures to support minimum requirements for hand hygiene in community settings. The total number of government measures identified per country is presented in the table along with their categorisation by Sanitation and Water for All (SWA) Building Blocks: SPS—sector policy strategy, IA—institutional arrangements, SF—sector financing, PMR—planning, monitoring, review, CD—capacity development. Indonesia is shown in the South-East Asia Region to maintain consistency with our analysis period; WHO reclassified Indonesia to the Western Pacific Region in May 2025.

The studies were conducted in 19 countries, with the majority coming from middle-income countries (71%, n=22). They spanned five of the six WHO regions: Africa (n=18), South-East Asia (n=8), Western Pacific (n=3), the Americas (n=1) and Europe (n=1), with no studies identified from the Eastern Mediterranean Region (figure 2). Most studies focused on government measures targeting household settings (58%, n=18), schools (19%, n=6) or both household and school settings (13%, n=4). One study covered measures for public spaces (markets), and two studies addressed measures for general community settings. Over half of the studies included measures targeting rural populations only (52%, n=16), while four studies (13%) included measures for both urban/peri-urban and rural populations. One study (3%) targeted urban populations exclusively, and the remaining studies did not specify whether the settings were urban or rural (32%, n=10). Targeted hand hygiene outcomes varied by study, with most focusing on changes in end-user hand hygiene practices alone or in combination with access to hand hygiene facilities (with soap and water).

Study quality scores ranged from 0 (no criteria met for the study’s methodological quality) to 5 (all criteria fully met for the study’s methodological quality). One quantitative descriptive study received a quality appraisal score of 0 due to the absence of clear research questions; however, it was retained in the systematic review because it provided valuable insights relevant to the research topic. Among the studies assessed (n=30), the overall mean quality score was 3.8, indicating generally good quality. The mean quality scores by study type were as follows: 3.9 for non-randomised studies (n=11), 3.8 for randomised control trials (n=8), 3.3 for quantitative descriptive studies (n=6) and 4.4 for mixed methods studies (n=5). Detailed quality appraisal results for each study are available in online supplemental material S3—MMAT Assessment.

### Evidence map of government measures to support hand hygiene in community settings

**Figure 3:**
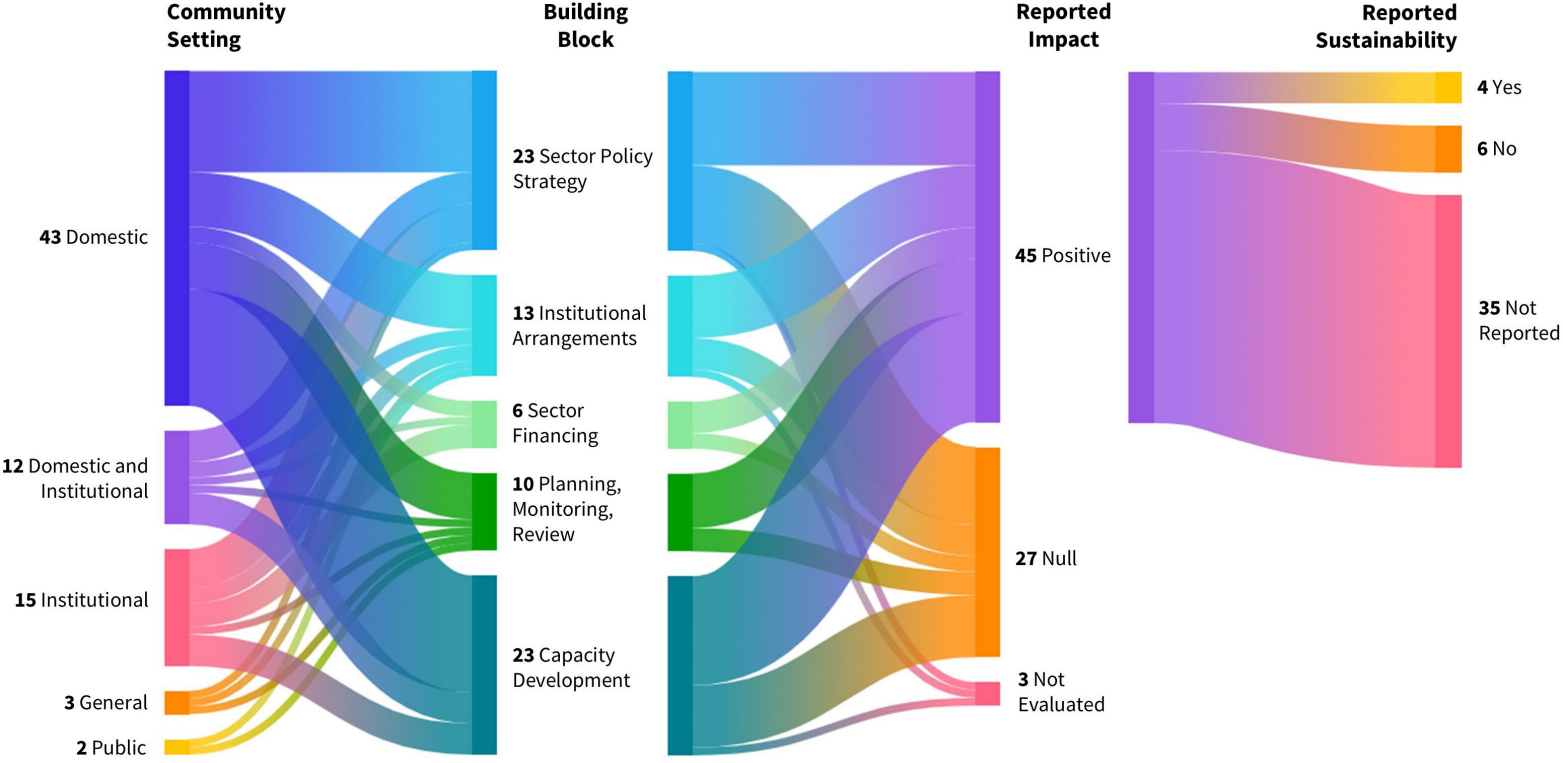
Evidence map of the relationships between community setting, the five Building Blocks, and reported impact on hand hygiene outcomes, along with reported sustainability of the impact. The thickness of lines represents the proportions of government measures (n=75). The colours show the grouping of government measures and trace the flow of measures between categories.

An evidence map of the relationships between community settings, the five Building Blocks and reported impact on targeted hand hygiene outcomes, along with reported sustainability of the impact, is presented in figure 3. Among the 31 included studies, 75 district government measures were identified and categorised into the five Building Blocks: sector policy strategy (31%, n=23), capacity development (31%, n=23), institutional arrangements (17%, n=13), planning, monitoring, review (13%, n=10) and sector financing (8%, n=6).

Over half of the studies (55%, n=17) reported positive impacts of government measures on the targeted hand hygiene outcomes. The remaining studies were associated with null impact (42%, n=13) or did not evaluate the government measures (3%, n=1). Among the identified government measures, 45 across all five Building Blocks were linked to 17 studies reporting a positive impact on hand hygiene outcomes. However, only four government measures were identified from studies that explicitly assessed or reported on sustainability (6%, n=2). These studies highlighted sustained improvements, such as access to handwashing facilities being maintained 2 years postimplementation^47^ and community health clubs continuing to function 14 years after the end of funding.^48^ Details on the extracted impact summary and sustainability details from each study are provided in online supplemental material S4—Summary of Studies.

### Thematic summary of government measures categorised into the five Building Blocks

A thematic summary of government measures categorised into the five Building Blocks and their reported impact on hand hygiene in community settings is presented in table 3. Seventeen descriptive themes of government measures were identified using a data-driven approach and definitions for each Building Block. Narrative themes for each Building Block are summarised in sections Sector policy strategy to Capacity development.

**Table 3:** Thematic summary of government measures (N=75) categorised into the five Building Blocks and their reported impact on hand hygiene in community settings

Building block	N (%)	Descriptive theme*	n (%)	Positive impact		Null impact		Not evaluated	
				Study ID	Average MMAT (n)	Study ID	Average MMAT (n)	Study ID	Average MMAT (n)
Sector policy strategy (SPS)	23 (31%)	**Programme integration†** Strategy for implementation which integrated hand hygiene into other sectoral government health programmes.	12 (52%)	Antwi-Agyei *et al*^54^ Bajracharya^55^ Christian *et al*^56^ Locks *et al*^57^ Mwakitalima *et al*^51^ Sanders *et al*^59^ Yeboah-Antwi *et al*^53^	4.0 (7)	Cameron *et al*^49^ Hernandez *et al*^50^ Newton-Lewis and Bahety^58^ Russpatrick *et al*^52^	3.8 (4)	Mugambi and Bery^60^	0.0 (1)
		**Promotion campaign†** Strategy for implementation which included hand hygiene-specific, government-led health programmes and campaigns utilising mass media and behaviour change communication strategies.	5 (22%)	Galiani *et al*^63^ Odone *et al*^65^ Quattrochi *et al*^64^	4.0 (3)	Briceño *et al*^61^ Chase and Do^62^	3.5 (2)	–	
		**Service delivery** Strategy for implementation which included service delivery approaches for the material requirements for hand hygiene.	4 (17%)	Chard and Freeman^47^	5.0 (1)	Graves *et al*^66^ Hasan and Gerber^68^ Schlegelmilch *et al*^67^	4.0 (3)	–	
		**Policy** Policies that identified goals and pathways to support hand hygiene practices.	2 (9%)	Yen *et al*^69^	5.0 (1)	Adebayo *et al*^70^	3.0 (1)	–	
Institutional arrangements (IA)	13 (17%)	**Committee and action planning†** Country-driven coordinating mechanisms which allowed for participation of a broad range of stakeholders in the planning for service delivery and sector learning around hand hygiene programming.	7 (54%)	Agarwala *et al*^71^ Ferguson *et al*^73^ Karon *et al*^74^ Quattrochi *et al*^64^ Waterkeyn and Cairncross^48^	4.2 (5)	Aluri *et al*^72^ Schlegelmilch *et al*^67^	3.0 (2)	–	
		**Cross-sector coordination** Country-driven coordinating mechanism which included the explicit engagement of actors across sectors within programmes aimed at promoting hand hygiene.	3 (23%)	Galiani *et al*^63^	4.0 (1)	Hernandez *et al*^50^	3.0 (1)	Mugambi 2013	0.0 (1)
		**Decentralising commitments†** Identification and allocation of institutional roles and responsibilities across ministries and departments.	2 (15%)	Antwi-Agyei *et al*^54^ Bajracharya^55^	3.5 (2)	–		–	
		**Legal and regulatory framework** Rules and regulations to govern and enforce compliance concerning hand hygiene initiatives.	1 (8%)	–		Lazaro *et al*^75^	3.0 (1)	–	
Sector financing (SF)	6 (8%)	**Financing** Identification of financing streams and responsibilities for sustained service delivery.	3 (50%)	Quattrochi *et al*^64^	4.0 (1)	Graves *et al*^66^ Hasan and Gerber^68^	4.5 (2)		
		**Resource allocation†** Strategic distribution and management of funds to support and implement programmes aimed at promoting and improving hand hygiene practices.	3 (50%)	Antwi-Agyei *et al*^54^ Karon *et al*^74^ Sanders *et al*^59^	4.0 (3)	–		–	
Planning, monitoring, review (PMR)	10 (13%)	**Monitoring and evaluation (M&E) systems** Formal planning, monitoring, and evaluation of sector performance which included systems to monitor hand hygiene indicators.	4 (40%)	Kavoo *et al*^76^ Sanders *et al*^59^	2.0 (2)	Hernandez *et al*^50^ Russpatrick *et al*^52^	3.5 (2)	–	
		**Accountability†** Mechanisms to ensure that responsible parties are held accountable which included routine monitoring visits by health officials and self-assessments within communities.	3 (30%)	Quattrochi *et al*^64^ Waterkeyn and Cairncross^48^	4.0 (2)	Lazaro *et al*^75^	3.0 (1)		
		**Learning†** Mechanisms for sector dialogue and learning review of sector performance through multi-stakeholder platforms.	3 (30%)	Agarwala *et al*^71^ Karon *et al*^74^ Mwakitalima *et al*^51^	3.7 (3)	–		–	
Capacity development (CD)	23 (31%)	**Implementer training** Capacity building and development through the training of implementers for hand hygiene promotion at scale, which included the use of community health worker/volunteer structures.	13 (57%)	Bajracharya^55^ Karon *et al*^74^ Locks *et al*^57^ Quattrochi *et al*^64^ Waterkeyn and Cairncross^48^ Yeboah-Antwi *et al*^53^	4.0 (6)	Aluri *et al*^72^ Briceño *et al*^61^ Chase and Do^62^ Graves *et al*^66^ Hernandez *et al*^50^ Koehn *et al*^77^	3.5 (6)	Mugambi and Bery^60^	0.0 (1)
		**Private sector engagement†** Plans that explicitly mentioned the engagement of the private sector for roles and responsibilities for sustainable service delivery at scale.	4 (17%)	Galiani *et al*^63^ Mwakitalima *et al*^51^ Yeboah-Antwi *et al*^53^	4.0 (3)	Cameron *et al*^49^	3.0 (1)	–	
		**Stakeholder/consumer engagement†** Plans that explicitly mentioned the engagement of sector stakeholders and individuals as educated consumers to support behaviour change and demand creation.	4 (17%)	Antwi-Agyei *et al*^54^ Galiani *et al*^63^ Mwakitalima *et al*^51^ Odone *et al*^65^	4.0 (4)	–		–	
		**Incentives** Provision of motivational rewards or benefits to institutions and individuals to enhance their participation in promoting hand hygiene outcomes.	2 (9%)	Ferguson *et al*^73^	5.0 (1)	Koehn *et al*^77^	4.0 (1)	–	

* Data-driven descriptive themes of identified government measures using definitions for each Building Block.

† Indicate themes in the Building Blocks where a majority of studies reporting on their application were associated with positive hand hygiene outcomes. MMAT-quality appraisal using the MMAT (Pluye and Hong^36^; Hong *et al*^37^): possible scores are 0–5 across study types (5=highest quality).

MMAT, Mixed Method Appraisal Tool.

#### Sector policy strategy

The sector policy strategy Building Block outlines policies and strategies for implementation, including agreements on implementation models and sustainable service delivery approaches (table 1). Most studies (74%, n=23) included a government measure categorised under sector policy strategy, which accounted for 31% (n=23) of the 75 extracted measures aimed at supporting equitable and sustained hand hygiene practices in community settings.

*Programme integration* was commonly identified as a strategy among the sector policy strategy government measures (52%, n=12). These measures involved integrating hand hygiene into other sectoral government health programmes, including sanitation (n=7),^49^,^55^ maternal and child health and nutrition (n=3),^56^,^58^ neglected tropical disease prevention and control (n=1)^59^ and HIV (n=1).^60^ The majority of these programme integration measures (58%, n=7) were associated with studies reporting a positive impact on the targeted hand hygiene outcomes (table 3).

*Promotion campaigns* included government-led health programmes and campaigns specifically focused on hand hygiene (ie, not integrated into other sectoral government health programmes). These campaigns utilised mass media and behaviour change communication strategies to promote and reinforce hand hygiene messaging.^61^,^63^ Some campaigns adopted a participant-initiated approach, where communities collectively requested the adoption of a programme,^64^ and kindergartens and primary schools opted into a national-level education programme during the COVID-19 pandemic.^65^ The majority of these promotion campaigns (60%, n=3) was associated with studies reporting a positive impact on the targeted hand hygiene outcomes (table 3).

*Service delivery* encompassed approaches for providing material requirements for hand hygiene, such as delivering WASH infrastructure in school settings (n=3)^47 66 67^ and piped water infrastructure to households with limited access to potable water (n=1).^68^ Among these service delivery measures, only one study reported a positive impact on the targeted hand hygiene outcomes^47^ (table 3).

*Policies* that set goals and pathways to support hand hygiene practices were at the national level (n=2). One policy recommended extending the use of ABHR beyond hospitals into broader communities during the 2009 H1N1 outbreak in Taiwan. This policy was associated with a study that reported a positive impact on hand hygiene outcomes.^69^ The other policy was a national school health policy in Nigeria, which outlined deliberate actions to ensure that schools are safe environments, including the provision of hand hygiene facilities. This policy was associated with a study that reported no positive impact on hand hygiene outcomes.^70^

#### Institutional arrangements

The institutional arrangements Building Block refers to formalised systems for governing and managing various aspects related to the WASH sector, including roles, responsibilities, coordination mechanisms and regulations (table 1). We identified several themes for institutional arrangements to support equitable and sustained hand hygiene practices in community settings, which represented 17% (n=13) of the measures identified in our review.

*Committee and action planning* was a commonly identified coordination mechanism among the institutional arrangements measures (54%, n=7). This mechanism involved the participation of a broad range of stakeholders—such as community members, government officials, water user associations, health club members, parents and teachers—in planning for service delivery and sector learning related to hand hygiene programming.^4864 67 71^,^74^ The majority of committee and action planning measures (71%, n=5) were associated with studies reporting a positive impact on targeted hand hygiene outcomes (table 3).

*Cross-sector coordination* was another identified coordinating mechanism that involved explicitly engaging actors across different sectors within programmes aimed at promoting hand hygiene. This was the second most common measure for institutional arrangements identified in our review (23%, n=3). Measures included engaging teachers to incorporate hand hygiene behaviour into the school curricula in Peru^63^; involving agricultural extension workers, model farmers and teachers as outreach agents for hygiene promotion in Ethiopia^50^; and inviting Acquired Immune Deficiency Syndrome (AIDs) and Sexually Transmitted Infection (STI) officers to trainings to support the integration of WASH and HIV programming in Kenya.^60^ The reported impact of these cross-sector coordination measures on hand hygiene outcomes was mixed (table 3).

*Decentralising commitments* concern identifying and allocating institutional roles and responsibilities across various ministries and departments. Both studies associated with these government measures reported positive impacts on the targeted hand hygiene outcomes (table 3).^54 55^

*Legal and regulatory framework* encompassed rules and regulations necessary for governing and enforcing compliance with hand hygiene initiatives. One study referenced a government measure related to this descriptive theme, which involved outlining by-laws for water availability at open-air markets for fresh fish vendors in Malawi.^75^ However, this measure was associated with a study that reported no positive impact on hand hygiene outcomes.

#### Sector financing

The sector financing Building Block refers to the allocation and management of financial resources (table 1). Sector financing was the least common government measure identified across studies (8%, n=6) and included themes on financing and resource allocation.

*Financing* included measures that focused on identifying financing streams and responsibilities for sustained service delivery (n=3).^64 66 73^ However, the reported impact of these measures on hand hygiene outcomes was mixed (table 3). Only one financing measure, which identified government support for new or improved water infrastructure and personnel costs, was associated with a study reporting positive impacts on targeted hand hygiene outcomes.^64^

*Resource allocation* involves measures related to the strategic distribution and management of funds to support and implement programmes aimed at promoting and improving hand hygiene practices. These measures included allocating a portion of school budgets for recurring expenses such as hygiene consumables and WASH infrastructure maintenance (n=2)^54 74^; and national-level prioritisation of funds to support service delivery (n=1).^59^ All three studies that identified government measures for resource allocation were consistently linked with positive impacts on hand hygiene outcomes (table 3).

#### Planning, monitoring, review

The planning, monitoring, review Building Block refers to the systematic monitoring and evaluation of sector performance, along with mechanisms for accountability and learning (table 1). We identified three themes within this Building Block, which represented 13% (n=10) of measures identified in our review.

*Monitoring and evaluation systems* included measures focused on formal planning, monitoring and evaluation of sector performance related to hand hygiene. Four studies across six African countries identified systems for monitoring hand hygiene indicators, making this the most common measure within the planning, monitoring, review Building Block (40%, n=4). These measures included developing country-specific indicators within a standard framework (F&E Monitoring and Evaluation framework) (n=1)^59^ and utilising volunteers and/or community workers to collect household-level data on hand hygiene (n=3).^50 52 76^ The reported impact of these measures on hand hygiene outcomes was mixed (table 3).

*Accountability* refers to mechanisms established to ensure that responsible parties are held accountable for their actions, decisions and performance concerning hand hygiene initiatives. Accountability measures included routine monitoring visits by health officials and self-assessments within communities related to hand hygiene behaviours.^48 64 75^ The majority of these accountability measures (67%, n=2) were linked with studies reporting a positive impact on targeted hand hygiene outcomes (table 3).

*Learning* refers to mechanisms for sector dialogue and the review of sector performance through multistakeholder platforms. Learning measures identified in the review included a multistakeholder platform (web-portal) to track contributions and monitor progress in the implementation of action plans across ministries and departments in India (n=1)^71^ and general experience sharing and dissemination efforts (n=2).^51 74^ All three studies that identified government measures related to learning within the planning, monitoring, review Building Block were consistently linked with positive impacts on hand hygiene outcomes (table 3).

#### Capacity development

The capacity development Building Block refers to plans that address the capacity of institutions, sector stakeholders and individuals (table 1). Most studies (61%, n=19) included a government measure categorised as capacity development, representing 31% (n=23) of the 75 extracted measures aimed at supporting equitable and sustained hand hygiene practices in community settings.

*Implementer training* was commonly identified as a strategy among measures for capacity development (57%, n=13). Governments used community health workers and volunteer structures to build capacity for hand hygiene promotion at scale. This included training hygiene promoters, health officials, health extension workers, community health workers, volunteers, teachers and community members.^4850 53 55 57 60^,^62 64 66 72 74 77^ However, most studies that identified implementer training measures were from studies with no reported impact on targeted hand hygiene outcomes (46%, n=6) or were not evaluated (8%, n=1) (table 3).

*Private sector engagement* refers to measures that explicitly involved the private sector in roles and responsibilities for sustainable service delivery at scale. Governments engaged with the private sector primarily in capacity building of local artisans and vendors to support the provision of hygiene products related to demand creation campaigns.^49 51 53 63^ The majority of private sector engagement measures (75%, n=3) was from studies that reported positive impacts on targeted hand hygiene outcomes (table 3).

*Stakeholder/consumer engagement* included measures that explicitly involved engaging sector stakeholders and individuals as educated consumers to support behaviour change and demand creation. These measures included the direct engagement with government officials and influential people^51^ and consumers^54 63 65^ to promote hand hygiene messaging. All four studies that identified government measures for stakeholder/consumer engagement were consistently linked with positive impacts on hand hygiene outcomes (table 3).

*Incentives* as capacity development involve providing motivational rewards or benefits to institutions and individuals to enhance their participation in promoting hand hygiene outcomes. This included performance-based incentives for local government bodies and health workers for achieving benchmarks related to hygiene practices.^73 77^ The reported impact of these incentive measures on hand hygiene outcomes was mixed (table 3).

### Government measures to address equality and/or affordability of handwashing

Government measures frequently targeted underserved or marginalised populations to address equity and protect community health. Half of the studies (52%, n=16) focused on rural populations, with 56% (n=9) reporting positive impacts on hand hygiene outcomes. Specific groups such as marginalised rural households,^68^ socioeconomically disadvantaged populations^55^ and people living with HIV and AIDS^60^ were addressed. Efforts to reach ‘hard-to-reach’ households included increased social mobilisation^55^ and providing health workers with bikes to travel to remote areas,^57^ both of which reported positive impacts on hand hygiene outcomes. These findings highlight the importance of tailoring measures to address the needs of underserved populations to enhance equity in hand hygiene initiatives.

Few measures specifically addressed the affordability of handwashing outside of those related to service delivery. However, several studies evaluated the effectiveness of demand-creation campaigns for hand hygiene, which often led to increased awareness and improved practices within communities. Among these, three studies referenced private sector engagement and training to support the construction of low-cost hand hygiene facilities, such as tippy taps.^50 51 61^ Of these studies, only one was associated with a positive impact on hand hygiene outcomes. These findings suggest that while direct affordability measures were limited, initiatives focusing on low-cost solutions and community-based training had potential, though their effectiveness varied.

## Discussion

This systematic review offers a comprehensive examination of government measures to support hand hygiene in community settings, analysing 31 studies and identifying 75 distinct measures. While over half of the studies reported positive impacts on hand hygiene practices, the findings highlight the importance of coordinated approaches across all WASH system components—policy, financing, institutional arrangements, planning, monitoring and capacity development—to achieve sustainable outcomes.

Across 9 of the 17 descriptive themes of government measures, evaluations more frequently reported positive results than null results. These themes, which span all five Building Blocks, underscore the challenge in pinpointing specific government measures that are consistently more impactful. Without adequate discussion of implementation details and contextual factors in the studies reviewed,^78^ we can describe *what* worked, but not *why* and *how* these government measures succeeded—nor how these findings can be applied across contexts. Given these limitations, we are cautious about making broad recommendations regarding which measures government should prioritise. Instead, we recommend that governments and policy stakeholders review studies relevant to the specific hand hygiene measures under consideration. Where possible, they should also engage with the study authors to ensure key lessons are appropriately adapted to the local context.

Most research to date has focused on policy strategy and capacity building, with limited attention to sector financing and implementation in public settings. While only a few evaluations of financing-related measures were identified, those included showed promising results. The majority of evaluations focused on domestic hand hygiene, with some addressing school-based interventions; however, few examined institutional and public settings more broadly. This review underscores both the challenges and the importance of evaluating complex government interventions, given the variation in outcomes across settings and the general lack of implementation detail. Enhancing standardised reporting and broadening the evidence base, particularly for public settings, will be essential to inform policy and improve community health resilience.^78 79^

The following sections summarise key trends identified through this systematic review, highlighting how government measures have been implemented and evaluated across diverse contexts.

### Government measures across all Building Blocks effectively supported hand hygiene in community settings

Sector policy strategy and capacity development were the most prevalent Building Blocks evaluated across various country contexts. Successful implementations of sector policy strategy included integrating hand hygiene into broader government health programmes^5153^,^57 59^ as well as hand hygiene-specific, government-led initiatives that utilised mass media and behaviour change communication to promote hand hygiene.^63^,^65^ Capacity development efforts, such as engaging with the private sector, stakeholders and consumers, were effective measures in driving behaviour change and creating demand for hand hygiene.^51 53 54 63 65^ Although sector financing was the Building Block least addressed in the literature, the studies that included measures for allocating resources consistently linked these measures with positive outcomes,^54 59 74^ indicating the need for more implementation and research in this area to develop applicable lessons learnt. Institutional arrangements and planning, monitoring, review Building Blocks were also associated with successful outcomes, emphasising the importance of committee development and action planning,^48 64 71 73 74^ decentralising commitments^54 55^ and establishing mechanisms for accountability and learning.^48 51 64 71 74^ While the evidence points to the potential of all Building Blocks to contribute to hand hygiene outcomes, it remains unclear which are the most impactful or should be prioritised in different contexts. Determining the relative influence of individual Building Blocks is inherently complex and under-researched, and future efforts should aim to better understand how these components interact, and which factors are most critical to enabling sustained hand hygiene improvements.

### Mixed results across government measures highlight the contextual barriers and challenges in evaluating multifaceted government initiatives and interventions

The high number of null results highlights challenges related to implementation, context-specific barriers and the inherent complexity of evaluating government initiatives and interventions at scale. The impacts of these government measures are by nature highly related to the context in which they are delivered. For example, while many governments employed community health workers and volunteer structures to build capacity and development for promoting hand hygiene at scale, the effectiveness of these approaches varied widely depending on the setting. In fact, most studies reporting on these measures showed either no measurable impact (46%) or were not evaluated (8%). The effectiveness of community health workers and volunteer structures, along with the factors influencing their impact, can vary significantly based on the local context.^80 81^

Additionally, while promotional campaigns were categorised under ‘sector policy strategy’, their effectiveness likely varies based on the modality of delivery and the behaviour change techniques used. A complementary systematic review on hand hygiene interventions found substantial heterogeneity in behaviour change techniques, with most interventions linked to education (eg, increasing knowledge or understanding), training (eg, imparting skills) and environmental restructuring (eg, changing the physical or social context).^30^ While the review found that a large proportion of identified interventions were effective, it also highlighted a misalignment between reported barriers and enablers and the intervention components implemented. This suggests that studies should either improve the reporting of known barriers and enablers or better leverage existing evidence in intervention design. This is particularly relevant for government measures, as policy decisions should be informed by evidence of which interventions align best with known barriers and enablers. Failing to address these factors can limit impact, while adding unnecessary components that may not be resource-efficient in terms of cost, time and effectiveness. Ensuring that government-led initiatives are designed with context-specific insights can enhance their effectiveness and sustainability, leading to greater public health impact.

Understanding why these measures succeeded or failed in improving hand hygiene requires additional detail on the implementation. Inconsistent reporting of WASH implementation presents a significant challenge in the sector,^79^ and the lack of detailed information on interventions further emphasises the need for improved reporting on WASH initiatives to better inform policy and programme design.^32^ Importantly, differences in positive and null results across the Building Blocks should be interpreted with caution, as these variations may reflect sectoral priorities or publication biases rather than the relative importance or effectiveness of specific components.

### Notable emphasis on government measures targeting household and school settings, with limited focus on public spaces

Approximately 75% of measures targeted either domestic (57%) or combined domestic/institutional (16%) settings, while only 3% of measures were specific to public settings. The COVID-19 pandemic prompted a wide array of national, regional and international initiatives aimed at improving hand hygiene.^11 25^ While this review provides insights into government measures for hand hygiene in households and schools, its applicability to diverse public and institutional settings such as markets, workplaces, public transportation and recreational areas is limited. These gaps in evidence are consistent with global monitoring efforts that primarily focus on WASH access in households^82^ and schools,^83^ leaving public settings and other institutional environments under-represented in hand hygiene interventions and assessments. The findings underscore the need to expand hand hygiene research and guidance in public settings, which could play a critical role in strengthening comprehensive preparedness and resilience in community health.

### Geographical limitations and gaps in global hand hygiene literature

Our review covered studies from only 19 countries, mostly in middle-income countries in Africa and Southeast Asia. Despite the crucial role of hand hygiene in preventing infectious diseases,^3^,^5^ there is a significant lack of comprehensive literature on government measures supporting hand hygiene in community settings. Given the limitations in the global literature, strengthening the findings of this systematic review could involve country-specific mapping of government policies and programmes through government stakeholders, including reports, policy documents and programme evaluations.^71^

### Strengths and Limitations

To our knowledge, this is the first systematic review to identify government measures to support hand hygiene in community settings organised according to an established framework. This review was part of an integrated protocol for multiple related reviews, which included an exhaustive search strategy encompassing multiple databases and grey literature sources and a two-phased approach to identify relevant literature of hand hygiene in community settings. A key strength of applying the SWA Building Blocks is its ability to provide a structured and comprehensive approach for evaluating and comparing government measures across diverse contexts. While the SWA Building Blocks primarily focus on water and sanitation service delivery, this framework is an extension of the WHO Building Blocks of Health Systems, which outline components that contribute to the strengthening of health systems in different ways.^41^ Using the SWA Building Blocks supported the generalisability of findings by offering a consistent structure for comparing and interpreting government measures across diverse studies and contexts. This is crucial for WASH, as it enables insights and strategies to be effectively applied and adapted across various settings.^31 32^ The trends identified underscore the need for a holistic approach to improving hand hygiene, showcasing a varied landscape of government interventions in terms of scope, implementation and impact.

Despite the thorough methodology employed in this systematic review, several limitations should be acknowledged. First, the inclusion of studies for this review was limited to those that identified the role of the government in measures taken to ensure effective hand hygiene within the title and abstract. Studies included in the review indicated government-led measures or measures led in collaboration with multilateral organisations (online supplemental material S5—Description of Government Measures). However, we note that robust reporting of implementation details, and the institutions who provided each intervention component, remains inconsistent in WASH studies.^79^ This limitation could have implications for the comprehensiveness of the evidence base reviewed. Furthermore, government measures to support hand hygiene often involve multifaceted interventions that address multiple aspects of the SWA Building Blocks simultaneously. Subjectivity is inherent in categorising interventions into discrete SWA Building Blocks and the related descriptive themes as it relies on researchers’ interpretation and judgement, which can introduce bias and inconsistency as reporting of intervention details varied across studies. Moreover, the heterogeneity of government measures, study methodologies and study designs presents a challenge to the synthesis and generalisability of findings. This was particularly true with respect to hand hygiene, as targeted outcomes varied by studies and typically included access to resources and individual behavioural practices, which are not mutually exclusive. Additionally, many studies lacked a clear control or comparison group, reflecting a common limitation in evaluating government programmes. To address this, we included a range of study designs. While this approach allowed for a more comprehensive assessment of available evidence, it also contributed to heterogeneity in study designs, measures and reported outcomes, further complicating synthesis and generalisability. While vote counting allowed for an overview of the distribution of positive and null findings, it does not account for effect sizes, study design or sample size and, therefore, may oversimplify complex findings. We acknowledge these limitations and interpret the vote-counting results with caution, using them primarily to highlight broad trends rather than to draw definitive conclusions about effectiveness.^84^ Finally, we only included studies that were published in English; thus, we may have missed insights from studies published in other languages.

## Conclusion

This systematic review highlights the diverse government measures implemented to support hand hygiene in community settings. The key findings indicate that sector policy strategy and capacity development were the most frequently reported measures, with mixed results across studies. While many government measures showed positive impacts, particularly those integrating hand hygiene into broader health programmes and employing multisectoral approaches, significant gaps remain in financing and implementation in settings beyond households and schools.

The findings suggest that governments should prioritise comprehensive policy frameworks that integrate hand hygiene promotion across multiple sectors, ensuring sustainability and equitable access. Institutional arrangements, such as cross-sector coordination and decentralised commitments, have demonstrated effectiveness in reinforcing hand hygiene interventions. However, challenges in resource allocation and long-term sustainability require targeted financial strategies.

For policymakers, these findings emphasise the need for structured governance mechanisms, investment in monitoring and evaluation systems and the inclusion of marginalised populations in hand hygiene initiatives. Practical recommendations include enhancing capacity development efforts through community health workers and private sector engagement, reinforcing regulatory frameworks and increasing funding for hand hygiene infrastructure in public spaces.

Future research should focus on evaluating the long-term sustainability of government-led hand hygiene interventions, assessing cost-effectiveness and exploring context-specific barriers to implementation. Comparative studies across different governance structures and country-income levels could provide deeper insights into best practices. Additionally, more research is needed on the effectiveness of hand hygiene interventions in public and high-risk community settings, such as markets and transport hubs, to guide targeted policy actions.

By addressing these evidence gaps, governments and practitioners can develop more effective, scalable and sustainable hand hygiene interventions that contribute to public health resilience and equitable access to hygiene resources.

## Supplementary material

10.1136/bmjgh-2025-018929 online supplemental file 1

10.1136/bmjgh-2025-018929 online supplemental file 2

10.1136/bmjgh-2025-018929 online supplemental file 3

10.1136/bmjgh-2025-018929 online supplemental file 4

10.1136/bmjgh-2025-018929 online supplemental file 5

## Data Availability

All data relevant to the study are included in the article or uploaded as supplementary information.

## References

[1] (2023). Burden of disease attributable to unsafe drinking-water, sanitation and hygiene: 2019 Update.

[2] Ross I, Esteves Mills J, Slaymaker T (2021). Costs of hand hygiene for all in household settings: estimating the price tag for the 46 least developed countries. BMJ Glob Health.

[3] Wolf J, Hubbard S, Brauer M (2022). Effectiveness of interventions to improve drinking water, sanitation, and handwashing with soap on risk of diarrhoeal disease in children in low-income and middle-income settings: a systematic review and meta-analysis. The Lancet.

[4] Ross I, Bick S, Ayieko P (2023). Effectiveness of handwashing with soap for preventing acute respiratory infections in low-income and middle-income countries: a systematic review and meta-analysis. The Lancet.

[5] Wolf J, Johnston RB, Ambelu A (2023). Burden of disease attributable to unsafe drinking water, sanitation, and hygiene in domestic settings: a global analysis for selected adverse health outcomes. The Lancet.

[6] Hutton G, Chase C (2017). Disease Control Priorities, Third Edition (Volume 7): Injury Prevention and Environmental Health.

[7] Esteves Mills J, Thomas A, Abdalla N (2023). How can global guidelines support sustainable hygiene systems?. BMJ Glob Health.

[8] Malteser International (2017). WASH guidelines for field practitioners: hygiene. https://www.malteser-international.org/fileadmin/dam/oeffentlich/malteser-international.de/Publikationen/Policies_and_Guidelines/MI_Water_Guidelines.pdf.

[9] UNICEF (2020). Understanding hygiene promotion in the context of risk communication & community engagement (RCCE) and infection control and prevention (IPC) for the covid-19 outbreak. https://www.corecommitments.unicef.org/kp/2020-04-covid-19-hygiene-programming-guidance-%28updated-01-apr%29---links-removed.pdf.

[10] WaterAid (2020). Technical guide for handwashing facilities in public places and buildings. https://washmatters.wateraid.org/publications/technical-guide-for-handwashing-facilities-in-public-places-and-buildings.

[11] WHO (2020). Water, sanitation, hygiene, and waste management for sars-cov-2, the virus that causes covid-19: interim guidance.

[12] WHO (2020). Actions for consideration in the care and protection of vulnerable population groups for covid-19. https://www.who.int/westernpacific/publications/m/item/WPR-DSE-2020-021-eng.

[13] GOAL (2020). WASH and infection prevention and control (ipc) measures in - households and public spaces. https://www.goalglobal.org/wp-content/uploads/2020/08/GOAL-COVID19-IPC-Community-and-HH.pdf.

[14] WHO (1986). Ottawa charter for health promotion.

[15] MacLeod C, Braun L, Caruso BA (2023). Recommendations for hand hygiene in community settings: a scoping review of current international guidelines. BMJ Open.

[16] WHO (2009). A guide to the implementation of the WHO multimodal hand hygiene improvement strategy.

[17] WHO (2009). WHO guidelines on hand hygiene in health care.

[18] WHO (2019). Water, sanitation and hygiene in health care facilities: practical steps to achieve universal access to quality care.

[19] WHO (2016). Guidelines on core components of infection prevention and control programmes at the national and acute health care facility level.

[20] WHO (2017). WHO recommendations on child health: guidelines approved by the who guidelines review committee.

[21] Sobsey MD, Water S (2002). Managing water in the home: accelerated health gains from improved water supply.

[22] WHO (2018). Guidelines on sanitation and health.

[23] MacLeod C, Esteves Mills J, Caruso BA (2024). Current international tools and guidance for the implementation of hand hygiene recommendations in community settings: a scoping review. Public and Global Health.

[24] Caruso BA, Snyder JS, Cumming O (2023). Synthesising the evidence for effective hand hygiene in community settings: an integrated protocol for multiple related systematic reviews. BMJ Open.

[25] Giné-Garriga R, Delepiere A, Ward R (2021). COVID-19 water, sanitation, and hygiene response: Review of measures and initiatives adopted by governments, regulators, utilities, and other stakeholders in 84 countries. Sci Total Environ.

[26] WHO (2022). Hand hygiene in communities and public settings: proposal for who guidelines. https://cdn.who.int/media/docs/default-source/wash-documents/hand-hygiene/concept-note-gl-on-hh-in-community-settings-with-logo.pdf?sfvrsn=c2140d3c_3.

[27] Caruso BA, Snyder JS, O’Brien LA (2025). Behavioural factors influencing hand hygiene practices across domestic, institutional and public community settings: a systematic review and qualitative meta-synthesis. BMJ Glob Health.

[28] O’Brien LA, Files K, Snyder JS (2025). Minimum material requirements for hand hygiene in community settings: a systematic review. BMJ Glob Health.

[29] Hilton SP, An NH, O’Brien LA (2025). Efficacy and effectiveness of hand hygiene-related practices used in community settings for removal of organisms from hands: a systematic review. BMJ Glob Health.

[30] Prasad SK, Snyder JS, LaFon E (2025). Interventions to improve hand hygiene in community settings: a systematic review of theories, barriers and enablers, behaviour change techniques and hand hygiene station design features. BMJ Glob Health.

[31] SWA (2016). Building blocks. sanitation and water for all (swa) secretariat. https://www.sanitationandwaterforall.org/about/our-work/priority-areas/building-blocks.

[32] Haque SS, Freeman MC (2021). The Applications of Implementation Science in Water, Sanitation, and Hygiene (WASH) Research and Practice. *Environ Health Perspect*.

[33] Page MJ, McKenzie JE, Bossuyt PM (2021). The PRISMA 2020 statement: an updated guideline for reporting systematic reviews. BMJ.

[34] Cooke A, Smith D, Booth A (2012). Beyond PICO:The SPIDER Tool for Qualitative Evidence Synthesis. Qual Health Res.

[35] (2023). Veritas health innovation. https://www.covidence.org/.

[36] Pluye P, Hong QN (2014). Combining the Power of Stories and the Power of Numbers: Mixed Methods Research and Mixed Studies Reviews. Annu Rev Public Health.

[37] Hong QN, Pluye P, Fàbregues S (2018). Mixed methods appraisal tool (MMAT) version 2018: user guide. Montr McGill Univ.

[38] WHO UN-water global analysis and assessment of sanitation and drinking-water (glaas). https://www.who.int/teams/environment-climate-change-and-health/water-sanitation-and-health/monitoring-and-evidence/wash-systems-monitoring/un-water-global-analysis-and-assessment-of-sanitation-and-drinking-water.

[39] Water UN (2020). The sdg 6 global acceleration framework. https://www.unwater.org/publications/sdg-6-global-acceleration-framework.

[40] Huston A, Moriarty P (2018). Understanding the WASH system and its building blocks.

[41] WHO (2010). Monitoring the building blocks of health systems.

[42] AMCOW (2021). African sanitation policy guidelines. https://amcow-online.org/african-sanitation-policy-guidelines-aspg/.

[43] VERBI Software (2021). MAXQDA 2022 [computer software]. https://www.maxqda.com.

[44] World Bank (2024). World bank country and lending groups. https://datahelpdesk.worldbank.org/knowledgebase/articles/906519-world-bank-country-and-lending-groups.

[45] Cooper HM, Rosenthal R (1980). Statistical versus traditional procedures for summarizing research findings. Psychol Bull.

[46] (1994). Handbook of Research Synthesis.

[47] Chard A, Freeman M (2018). Design, Intervention Fidelity, and Behavioral Outcomes of a School-Based Water, Sanitation, and Hygiene Cluster-Randomized Trial in Laos. IJERPH.

[48] Waterkeyn J, Cairncross S (2005). Creating demand for sanitation and hygiene through Community Health Clubs: A cost-effective intervention in two districts in Zimbabwe. Soc Sci Med.

[49] Cameron L, Shah M, Olivia S (2013). The world bank.

[50] Hernandez O, Rosenbaum J, Faris K (2011). Combining sanitation and hand washing promotion: an example from Amhara. Ethiopia.

[51] Mwakitalima A, Massa K, Seleman A (2018). Scaling up rural sanitation in Tanzania: evidence from the National Sanitation Campaign. J Water Sanit Hyg Dev.

[52] Russpatrick S, Tiwari A, Markle L (2017). Mobility up the sanitation ladder following community-led total sanitation in rural Zambia. J Water Sanit Hyg Dev.

[53] Yeboah-Antwi K, MacLeod WB, Biemba G (2019). Improving Sanitation and Hygiene through Community-Led Total Sanitation: The Zambian Experience. Am J Trop Med Hyg.

[54] Antwi-Agyei P, Mwakitalima A, Seleman A (2017). Water, sanitation and hygiene (WASH) in schools: results from a process evaluation of the National Sanitation Campaign in Tanzania. J Water Sanit Hyg Dev.

[55] Bajracharya D (2003). Myanmar experiences in sanitation and hygiene promotion: lessons learned and future directions. Int J Environ Health Res.

[56] Christian P, Hurley KM, Phuka J (2020). Impact Evaluation of a Comprehensive Nutrition Program for Reducing Stunting in Children Aged 6–23 Months in Rural Malawi. J Nutr.

[57] Locks LM, Nanama S, Addo OY (2019). An integrated infant and young child feeding and small‐quantity lipid‐based nutrient supplementation programme in the democratic republic of congo is associated with improvements in breastfeeding and handwashing behaviours but not dietary diversity. Matern Child Nutr.

[58] Newton-Lewis TA, Bahety G (2021). Evaluating the effectiveness of Community Health Worker home visits on infant health: A quasi-experimental evaluation of Home Based Newborn Care Plus in India. J Glob Health.

[59] Sanders AM, Dixon R, Stuck L (2021). Evaluation of facial cleanliness and environmental improvement activities: Lessons learned from Malawi, Tanzania, and Uganda. PLoS Negl Trop Dis.

[60] Mugambi E, Bery R (2013). Promoting healthy hygiene and sanitation practices for people living with HIV and AIDS.

[61] Briceño B, Coville A, Gertler P (2017). Are there synergies from combining hygiene and sanitation promotion campaigns: Evidence from a large-scale cluster-randomized trial in rural Tanzania. PLoS One.

[62] Chase C, Do Q-T (2012). The world bank.

[63] Galiani S, Gertler P, Orsola-Vidal A (2012). Promoting Handwashing Behavior in Peru: The Effect of Large-Scale Mass-Media and Community Level Interventions.

[64] Quattrochi JP, Coville A, Mvukiyehe E (2021). Effects of a community-driven water, sanitation and hygiene intervention on water and sanitation infrastructure, access, behaviour, and governance: a cluster-randomised controlled trial in rural Democratic Republic of Congo. BMJ Glob Health.

[65] Odone A, Bricchi L, Signorelli C (2021). COVID-19 control school-based interventions: characteristics and impact of a national-level educational programme in Italy. Acta Bio Medica Atenei Parm.

[66] Graves JM, Daniell WE, Harris JR (2012). Enhancing a Safe Water Intervention with Student-Created Visual AIDS to Promote Handwashing Behavior in Kenyan Primary Schools. Int Q Community Health Educ.

[67] Schlegelmilch MP, Lakhani A, Saunders LD (2016). Evaluation of water, sanitation and hygiene program outcomes shows knowledge-behavior gaps in Coast Province, Kenya. Pan Afr Med J.

[68] Hasan MM, Gerber N (2016). The Impacts of Piped Water on Water Quality, Sanitation, Hygiene and Health in Rural Households of North-Western Bangladesh - A Quasi-Experimental Analysis. SSRN Journal.

[69] Yen M-Y, Yen Y-F, Chen S-Y (2021). Learning from the past: Taiwan’s responses to COVID-19 versus SARS. Int J Infect Dis.

[70] Adebayo AM, Dania O, Akindele A (2021). Public–private comparative study of the quality of implementation of the school health programme in a metropolitan city in Nigeria. J Public Health (Berl).

[71] Agarwala T, Shiva R, Saha S (2022). Mapping policies and tracking budgets for hand hygiene: a study of select departments in Odisha.

[72] Aluri KZ, Halder AK, Islam M (2022). The effect of a large‐scale water, sanitation and hygiene intervention in Bangladesh on knowledge, behaviour and health: Findings from an endline programme evaluation. *Tropical Med Int Health*.

[73] Ferguson L, Boudreaux C, Cheyassin Phall M (2022). Facility and Community Results-Based Financing to Improve Maternal and Child Nutrition and Health in The Gambia. Health Systems & Reform.

[74] Karon AJ, Cronin AA, Cronk R (2017). Improving water, sanitation, and hygiene in schools in Indonesia: A cross-sectional assessment on sustaining infrastructural and behavioral interventions. Int J Hyg Environ Health.

[75] Lazaro J, Kapute F, Holm RH (2019). Food safety policies and practices in public spaces: The urban water, sanitation, and hygiene environment for fresh fish sold from individual vendors in Mzuzu, Malawi. Food Sci Nutr.

[76] Kavoo DM, Ali SH, Kihara AB (2016). An assessment of water, sanitation and hygiene (wash) practices and quality of routinely collected data in Machakos County Kenya. East Afr Med J.

[77] Koehn HJ, Zheng S, Houser RF (2020). Remuneration systems of community health workers in India and promoted maternal health outcomes: a cross-sectional study. BMC Health Serv Res.

[78] Crocker J, Ogutu E, Snyder JS (2024). TIDieR-WASH: A Guideline for Reporting Implementation of Water, Sanitation, and Hygiene Interventions. Environ Health Perspect.

[79] Crocker J, Ogutu EA, Snyder J (2024). The state of reporting context and implementation in peer-reviewed evaluations of water, sanitation, and hygiene interventions: A scoping review. Int J Hyg Environ Health.

[80] Kane S, Kok M, Ormel H (2016). Limits and opportunities to community health worker empowerment: A multi-country comparative study. Soc Sci Med.

[81] Ahmed S, Chase LE, Wagnild J (2022). Community health workers and health equity in low- and middle-income countries: systematic review and recommendations for policy and practice. Int J Equity Health.

[82] WHO (2021). Progress on household drinking water, sanitation and hygiene 2000-2020: five years into the sdgs.

[83] WHO (2022). Progress on drinking water, sanitation and hygiene in schools: 2000-2021 data update.

[84] Higgins JPT, Thomas J, Chandler J (2019). Cochrane handbook for systematic reviews of interventions.

