# Supplementary figures and images for: Effectiveness of measures taken by governments to support hand hygiene in community settings: a systematic review
Source: BMJ Glob Health. 2025 Sep 16;10(Suppl 7):e018929. doi: 10.1136/bmjgh-2025-018929 (PMC12443189; doi:10.1136/bmjgh-2025-018929)

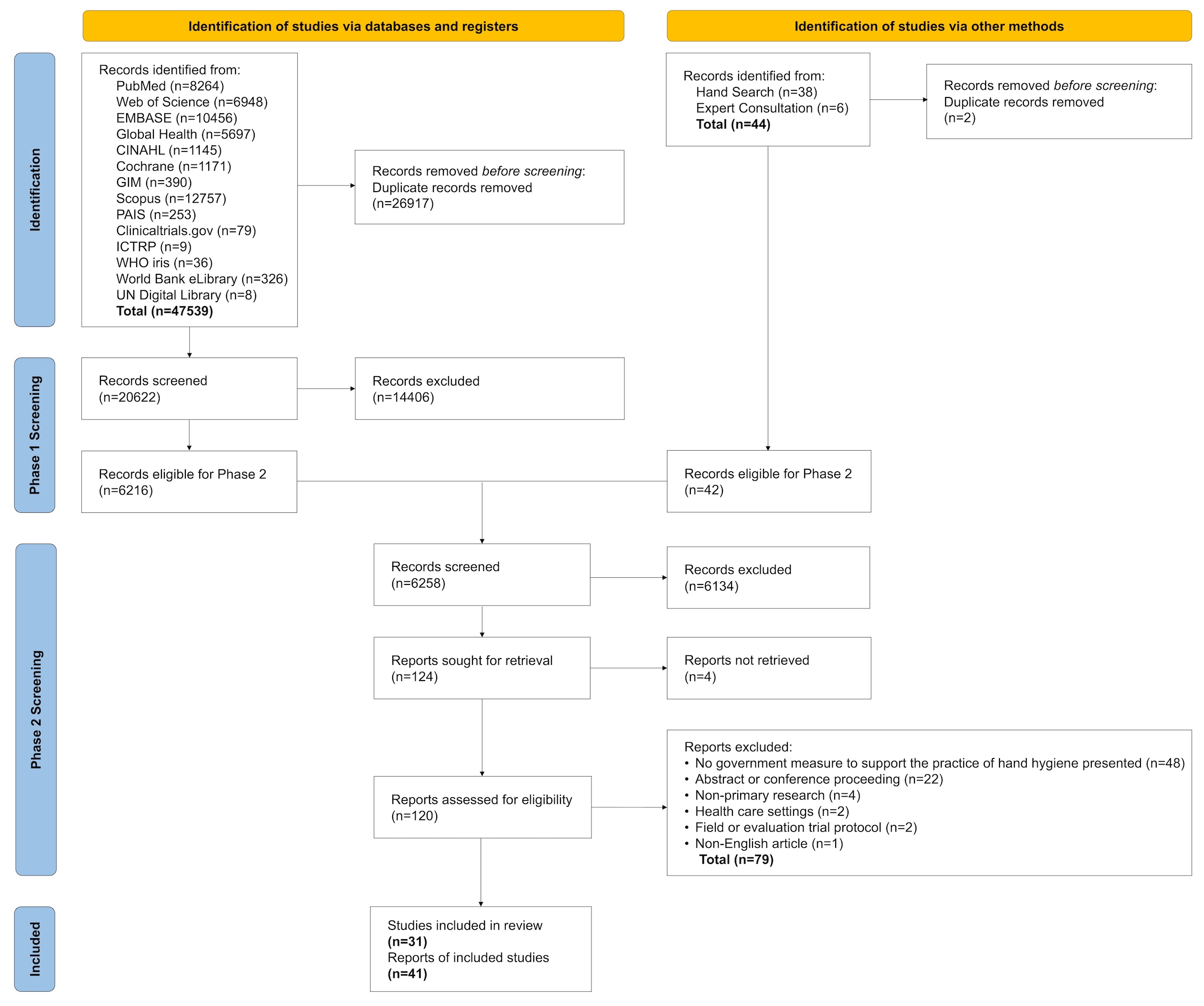

Supplement: online supplemental file 2 [file bmjgh-10-Suppl_7-s002.jpg]
